# Ethical and secure evidence generation from regionwide clinical data through a collaborative environment for advancing predictive care

**DOI:** 10.3389/fpubh.2025.1630351

**Published:** 2025-08-08

**Authors:** Dolores Muñoyerro-Muñiz, Román Villegas, Víctor de la Oliva, Alberto Esteban-Medina, Patricia Fernández del Valle, Ana Sánchez, M. Belen Susin, Isidoro Gutierrez-Alvarez, Marta Reboredo, Laura Alejos, Carlos Loucera, Joaquín Dopazo

**Affiliations:** ^1^Subdirección Técnica Asesora de Gestión de la Información, Servicio Andaluz de Salud, Sevilla, Spain; ^2^Andalusian Platform for Computational Medicine, Andalusian Public Foundation Progress and Health-FPS, Seville, Spain; ^3^R&I Centers, Programs and Projects Management Area, Andalusian Public Foundation Progress and Health-FPS, Seville, Spain; ^4^Institute of Biomedicine of Seville (IBiS), University Hospital Virgen del Rocío/CSIC/University of Sevilla, Sevilla, Spain

**Keywords:** real-world data, electronic health records, predictive medicine, secure processing environment, artificial intelligence, data privacy

## Abstract

Ensuring data protection is a major challenge in clinical research involving sensitive patient information. However, secure processing environments (SPEs) enable the ethical and compliant secondary use of real-world data (RWD) for evidence generation. This study presents a collaborative infrastructure integrating a comprehensive Health Population Database (BPS) with a legal and computational framework to facilitate secure, large-scale clinical studies. The Andalusian Platform for Medical Evidence Generation is an SPE embedded within the Andalusian healthcare network, leveraging RWD from over 15 million patients from the BPS. It supports diverse studies, including treatment efficacy, survival analyses, and predictive modeling, while ensuring alignment with the General Data Protection Regulation (GDPR) and proactively designed to meet forthcoming European Health Data Space (EHDS) requirements. Data are processed within a secure ecosystem, preventing unauthorized access and enabling legally compliant research collaborations. By combining clinical RWD with a robust ethical and legal framework, we present a scalable model for secure, data-driven region-level healthcare innovation. The platform supports cost-effective predictive models, particularly relevant for aging populations, and establishes a blueprint for regional and international adaptation. This approach strengthens the role of healthcare systems in both knowledge generation and sustainable economic growth, ensuring that patient data is leveraged for scientific and societal benefit.

## 1 Introduction

The use of Real-World Data (RWD) is transforming healthcare research by enabling large-scale studies on treatment effectiveness, disease progression, and healthcare resource utilization in routine clinical settings (1). Unlike randomized controlled trials (RCTs), which have strict inclusion criteria and often exclude diverse patient populations, RWD provides a broader and more representative perspective on real-world clinical outcomes (2). By using RWD from electronic health records (EHRs), hospital admissions, pharmacy records, laboratory tests, and disease registries, researchers can generate Real-World Evidence (RWE) that supports clinical decision-making, regulatory policies, and medical innovation (3, 4).

One of the most significant advantages of RWD is its ability to accelerate research and improve patient outcomes by providing insights that complement traditional clinical trials (5). This is particularly valuable in contexts where RCTs are impractical, such as rare diseases, where patient populations are small, or during public health emergencies, like the COVID-19 pandemic, where rapid evidence generation is essential (6, 7). Moreover, RWD plays a crucial role in post-marketing drug surveillance (Phase IV studies), enabling the continuous assessment of drug safety and effectiveness in broader patient populations (8). Also, an upsurge in preventive medicine is foreseeable from the possibility of deriving early condition, diagnosis or end-point predictors from retrospective RWD studies (9, 10).

Despite its potential, the use of RWD presents several challenges, particularly regarding data privacy, security, and regulatory compliance. Ensuring that sensitive patient information is protected while enabling meaningful research is a key priority in modern healthcare. The European Health Data Space (EHDS) (11) aims to establish a legal framework that facilitates the secure and ethical use of health data across Europe while complying with the General Data Protection Regulation (GDPR) (12).

In this context, the Andalusian Health Population Database (BPS, acronym for “Base Poblacional de Salud” in Spanish) stands as one of the most comprehensive health data repositories in Europe, containing EHRs from over 15 million patients since 2001 (13). The Andalusian Platform for Medical Evidence Generation (PAGEM, from its acronym in Spanish), is an instrumental infrastructure, hosted within the Andalusian Public Health System, that provides a Secure Processing Environment (SPE) that enables the secondary use of this data for research while ensuring compliance with GDPR and EHDS regulations (14). Through this infrastructure, researchers can conduct treatment efficacy studies, survival analyses, and predictive modeling while maintaining strict data security standards, ensuring patient data privacy.

This paper presents PAGEM as a model for ethical and secure RWD utilization, demonstrating its potential to shift healthcare from a reactive to a predictive approach. By ensuring data protection, regulatory compliance, and research accessibility, PAGEM exemplifies how public health systems can leverage clinical big data to drive medical innovation and improve patient outcomes, setting a standard for other regions and countries to follow within the new EHDS.

## 2 Material and equipment

### 2.1 A region-wide collaborative environment for secure and ethical generation of evidence from medical data

A region-wide collaborative environment for secure and ethical secondary use of medical data to generate medical evidence has been structured around three fundamental components: first, the PAGEM, a SPE with a state-of-the-art computing infrastructure that ensures data security and confidentiality for secondary use of medical data for research purposes; second, an immense medical data lake, the BPS, integrating EHRs and other clinical data from over 15 million patients, covering a wide range of health indicators, diagnoses, treatments, and healthcare utilization; and third, a regional legal and ethical framework, compliant with GDPR and the EHDS, designed to ensure that patient privacy is protected while enabling the responsible use of information for research purposes.

Within this environment, researchers work within the healthcare corporate network, where data never leaves the controlled secure environment. This privacy-preserving model eliminates risks associated with data transfers while enabling high-performance analytics, including artificial intelligence (AI)-driven studies, survival analyses, drug safety assessments, etc.

### 2.2 The PAGEM infrastructure

The PAGEM is a SPE consisting of a computational infrastructure initially funded within the scope of the Andalusian Plan for Research, Development and Innovation (14). This infrastructure is located within the SSPA corporate network and has specifically conceived for the ethical and secure analysis of data protected by the GDPR. PAGEM is compliant with the definition of SPE as described in Article 50 of the Resolution of the European Parliament of 24 April 2024 on the proposal for a Regulation of the European Parliament and of the Council on the EHDS (11).

#### 2.2.1 Open software, reproducibility and explainability

For the sake of reproducibility and explainability, all the software used in the studies carried out in the PAGEM SPE is open. For data processing, Python (15) and its extensive ecosystem of libraries, such as Pandas (16) and NumPy (17) is used. For statistical analysis, Python libraries like Numpy, Scipy (18) and statsmodels (19) are used, whereas R (20) and Bioconductor (21) packages are used for some advanced analysis. AI-driven models, including machine learning and deep learning, are backed by the core libraries of the Python scientific distribution, like NumPy and SciPy, as well as specialized tools like scikit-learn (22), and TensorFlow (23).

To ensure computational reproducibility, software versioning is achieved using locally managed gitlab servers. Conda environments, based on the conda-forge (24) and Bioconda (25) channels, provide project-specific software dependencies. Additionally, entire analysis environments are encapsulated within Docker containers (26). Finally, interactive analyses are facilitated through securely-served Jupyter notebooks (27), leveraging the aforementioned reproducibility infrastructure for computation.

#### 2.2.2 Hardware

The PAGEM hardware infrastructure is specifically designed to meet the rigorous computational demands of modern clinical Big Data research. A general-purpose computing cluster provides significant computational capacity, currently featuring 1,832 cores, 13,568 GB of RAM, and 993 TB of storage. For deep learning applications, a specialized GPU server cluster is available to handle the high-performance requirements of AI-driven research. This cluster includes 192 CPU cores, 4,864 GB of RAM, and 18 high-performance GPUs.

### 2.3 The BPS, a regionwide medical data lake

The Andalusian Public Health System (SSPA from its acronym in Spanish) provides service to the population of the Andalusia region (Southern Spain), with a population of ~8.5 million inhabitants, and comprises a total of 55 hospitals and 34 primary care districts with a total of 1,505 primary care centers. The SSPA is fully digitalized by means of the digital system Diraya, where all the data are indexed and referred to the patient. Diraya incrementally dumps all patient data in the BPS on a monthly basis. The BPS is a health information system that collects clinical data and data on the use of health resources of each person receiving health care in the SSPA, totalling over 15 million patients accumulated since 2001. To illustrate the dimension of BPS it is worth mentioning some figures (see Table 1) about the data stored, such as the 874 million diagnoses, the 2,428 million analytical tests, or the 7,000 million medical images, to cite just a few examples. These data originate from different professional sources within the health system: medical diagnoses are typically entered by physicians; nursing diagnoses by trained nursing personnel; laboratory results by automated systems validated by clinical laboratory staff; and imaging records by radiology services. Each category of data serves a distinct clinical purpose, from initial diagnosis and monitoring to treatment planning and follow-up care. This volume makes BPS one of the largest medical RWD repositories in the world.

**Table 1:** BPS in figures.

**Data type**	**Source**	**Data**	**Number (millions)**
		Users/Patients (2001–September 2024)	15.8
Total users	Administrative	Active users (2024)	8.89
		Men (2024)	4.51
		Women (2024)	4.38
	Medical doctors	Medical diagnoses (90% automatic coding)	874.1
	Nursing staff	Nursing diagnoses	51.3
Clinical	Laboratory staff	Analytical test results	2,428
practice	Medical image staff	PACS images	7,000
	Medical doctors	Vital signs (weight, height, BMI, blood pressure)	83.5
	Nursing staff	Functional evaluations	4.2
	Nursing staff	Cognitive assessments	2.7
		Vaccination events	79
Processes	Administrative	Prosthesis and implant records	0.7
		Temporary Incapacity (TI) processes	11.2
		Hospital stays	3.5

From the data collected in BPS, it is possible to obtain estimates on health, the behavior of patients and, in general, users in relation to health services and to stratify the population in order to guide the provision of these services. The BPS also enables longitudinal studies to be carried out, the incidence of pathologies to be estimated and projections to be made on the state of health of the population and its resource needs. It also makes it possible to analyse efficiency in the use of resources by health care providers. Since its inception, BPS was conceived with a strong focus on research. In fact, it has been included in the Repository of Innovative Practices in Active and Healthy Aging of the European Commission (28).

The structure of BPS is defined in a reference publication (13), which describes the development of this information system that connects data from multiple Electronic Health Records (EHR) to improve assistance to patients, health services administration, management, evaluation, and inspection, as well as public health and research. BPS connects pseudonymized data from nearly any individual of the whole Andalusia population (13). The data are sourced from different SSPA information systems including: EHRs, the minimum basic data sets (inpatients, outpatient major surgery, hospital emergencies and medical day hospital), mental health information systems, analytical and image tests, vaccines, renal patients, and pharmacy, among others. In order to have the data as structured as possible, an automatic coder developed in-house for hospital emergency and primary care electronic medical records (29) is used to code clinical diagnoses into ICD10. Also 80 chronic pathologies were identified and coded. Table 2 presents a selection of prevalent chronic and high-impact conditions as coded in BPS. These include both non-communicable diseases (e.g., diabetes, cardiovascular disorders) and cancers. The list has been produced by BPS curators based on prevalence, impact on health services, and standardized ICD-10 coding coverage (13).

**Table 2:** Some prevalent diseases in BPS.

**Pathology**	**Women**	**Men**	**Total**
Dyslipemia	1,085,213	966,250	2,051,463
Hypertension	1,018,136	915,329	1,933,465
Arthrosis, spondylosis	979,136	557,983	1,537,119
Anxiety disorder	698,438	329,416	1,027,854
Asthma	511,843	441,215	953,058
Diabetes	380,105	414,329	794,434
Hypothyroidism	596,937	130,390	727,327
Colorectal cancer	27,079	33,271	60,350
Lung cancer	5,050	10,525	15,575
Breast cancer	96,171	2,107	98,278
Heart failure	120,430	106,849	227,279
Atrial fibrillation	86,311	91,836	178,147

Cases in 2023.

To ensure data quality and harmonization, the BPS relies on a standardized, patient-indexed structure supported by Diraya, the unified digital health system. As previously mentioned, diagnostic information is coded using ICD-10, with automatic coders improving consistency across care levels. Other standard codes are used, such as Anatomical Therapeutic Chemical (ATC) Classification for drugs (30). Data from diverse sources (EHRs, pharmacy, imaging, lab tests, etc.) are integrated through validated linkage processes and updated monthly. Each data field undergoes validation checks, and the system maintains full traceability. For participation in international projects, data can be mapped to standardized structures such as the OMOP Common Data Model, facilitating semantic interoperability and federated analytics.

The BPS also supports linkage to external datasets, such as biobank-derived genomic data, clinical trial cohorts, registries, survey results, etc., provided the integration is justified, ethically approved, and technically feasible via pseudonymized identifiers. This enables enriched, longitudinal research studies that combine clinical, molecular, and behavioral dimensions, further expanding the research potential of the platform.

## 3 Methods

### 3.1 Secure data management in PAGEM

The PAGEM infrastructure complies with the GDPR and adheres to the Joint Resolution 1/2021 of the General Secretariat for Research, Development and Innovation in Health of the Regional Ministry of Health and Families and the Management Directorate of the Andalusian Health Service (31), which anticipates key elements of the forthcoming EHDS regulation (11). Specifically, it follows the principles of lawfulness, fairness, transparency, purpose limitation, data minimization, and integrity/confidentiality as defined in Articles 5–9 of the GDPR. Data processing is grounded in Article 6(1)(e) (task carried out in the public interest) and Article 9(2)(j) (scientific research purposes). Although the EHDS regulation is not yet finalized, the PAGEM SPE is aligned with its current legislative resolution (24 April 2024), particularly Article 50, and is proactively designed to fulfill its anticipated technical and governance criteria for Secure Processing Environments

Since the infrastructure is located within the SSPA corporate network and is operated by personnel of the Foundation Progress and Health, which belongs to the health system, data never leave the secure environment of the health system and are managed by trusted personnel from the health system. These two aspects are crucial for the Data Protection Impact Assessment (DPIA), given that the relative risk for the data used in the study is minimized (32). The DPIA is a document consisting of a description of the data life cycle, detailing the activities to be carried out, the specific data to be processed and the people and technologies involved, both for the data acquisition process and for its storage, processing, transfer to third parties and final destruction. The DPIA includes the description of potential hazards, their inherent and residual risk, and mitigation measures that could be implemented. By analyzing the data in the PAGEM infrastructure, an action plan is not required (32).

**Figure 1:**
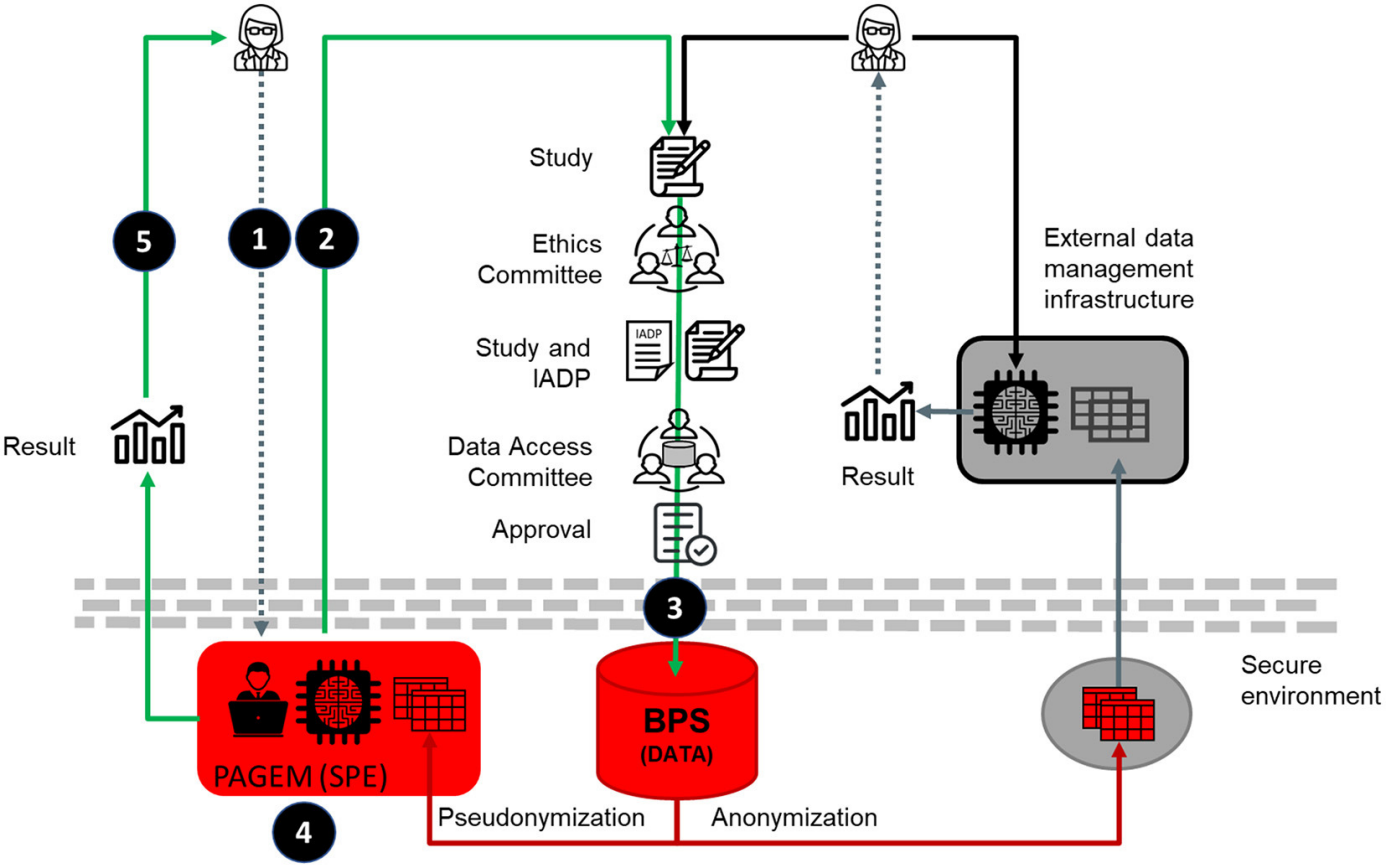
Schematic representation of the collaborative environment for secure and ethics data analysis. The left side illustrates the PAGEM model, in which only authorized health system personnel analyse pseudonymized data within the SPE, and researchers receive only aggregated results. This contrasts with potential EHDS-compliant models (right side), which may support controlled researcher access to pseudonymized individual-level data within SPEs. Schematically, 1) the researcher agrees a study with the PAGEM, 2) the PAGEM takes care of the permissions for data management and, 3) once granted, the PAGEM request to BPS the extraction of pseudonymized data, 4) the PAGEM team and, occasionally researchers with a DPA, perform the analysis and 5) the results, that does not contain any private data, are released. The right side of the figure represents the conventional data analysis procedure.

Summarizing, the data management procedure (described in the DPIA as the life cycle of the data and schematised in Figure 1) is as follows: i) once the study is approved by the data access committee the PAGEM work team request the data to BPS, ii) the BPS team extract the data and pseudonymize it, iii) the BPS transfer the pseudonymized data to the PAGEM infrastructure, iv) the PAGEM work team carries out the analysis of the data as described in the research protocol, v) once is done the data is removed from the PAGEM infrastructure.

It is important to clarify that the Joint Resolution 1/2021 requires that the principal researcher (or one of them, if there are more than one), belongs to the Andalusian health System. Therefore, in the current configuration of PAGEM, only authorized personnel from the health system (i.e., members of the Andalusian Health System, including the PAGEM work team, and occasionally other researchers with a data processing agreement -DPA-) access and analyse the pseudonymized individual-level data within the SPE. Researchers external to the health system receive only aggregate, non-identifiable results. This conservative data governance model prioritizes privacy and aligns with national interpretations of GDPR and regional ethical oversight. However, the EHDS regulation supports a broader model where accredited researchers can be granted direct access to pseudonymized individual-level data within SPEs under strict safeguards. While PAGEM does not yet implement this model, it has been technically and procedurally designed to allow for such evolution in the future, once regulatory and governance adjustments permit.

The use of pseudonymized data is an important asset specific to this collaborative environment (Figure 1 left part), as it allows patient re-identification when necessary. This approach contrasts with many conventional data analysis frameworks (Figure 1, right side), which can also use pseudonymized data but typically restrict or preclude any mechanism for re-identification, even under authorized circumstances, by anonymizing the data. In contrast, the PAGEM model preserves the ability to re-identify patients when ethically justified and approved by the ethics committee, a key enabler for studies that may trigger clinical action or benefit.

### 3.2 Regional regulation for ethical and secure data access

The most recent regulation for the use of medical data for research purposes in the Andalusia region was issued the 4th December, 2021, in the Joint Resolution 1/2021 of the General Secretariat for Research, Development and Innovation in Health of the Regional Ministry of Health and Families and the Management Directorate of the Andalusian Health Service (31). This innovative resolution anticipated the procedure for access to medical data which has further been described in the recent European Parliament legislative resolution (Regulation 2025/327, OJ L 2025/327, 5 March 2025) in the proposal for a Regulation of the European Parliament and of the Council on the EHDS (11). According to such Regulation, primary use refers to the processing of health data for the direct provision of healthcare services, whereas secondary use includes activities such as scientific research, public health planning, and health policy development. The PAGEM framework is designed specifically for secondary use, governed by ethical approval and strict safeguards for privacy and data minimization.

The Joint Resolution 1/2021 defines a Health Data Access Body (Data Access Committee, DAC) responsible for granting access to medical data for secondary use. In order to evaluate the appropriateness and justification of the request of access to the data based on scientific, ethical, and legal criteria, the DAC requires: i) the research protocol for which the data is requested, ii) the permission of the Coordinating Committee on Biomedical Research Ethics of Andalucía (CCEIBA) (33), iii) the DPIA (32) document, subject to the GDPR and taking into account the Horizon 2020 Programme Guidance How to complete your ethics self-assessment (34) and, iv) signed commitment of the principal researcher in which he/she undertakes not to redistribute data, not to attempt to re-identify individuals in the dataset and removing the dataset once the study has finished. Typically, one of the members of the PAGEM work team acts as co-principal researcher in the study protocols and takes the responsibility of comply with all the commitments.

The secondary use of clinical data for research purposes in PAGEM is based on Articles 6(1)(e) and 9(2)(j) of the GDPR, which allow data processing when necessary for tasks in the public interest and scientific research, respectively, provided appropriate safeguards are implemented. This legal basis is explicitly recognized under Spanish national law, in which the Seventeenth Additional Provision of Organic Law 3/2018 on the Protection of Personal Data and Guarantee of Digital Rights (LOPDGDD) (35) does provide for an exemption from informed consent in certain research contexts, especially when large databases are used and it is not possible to provide individualized information to data subjects because it would be a disproportionate effort.

Additionally, this legal basis is operationalized through the regional Joint Resolution 1/2021, which defines the procedures and oversight mechanisms, including the mandatory review by an accredited ethics committee (CCEIBA), approval by a Data Access Committee (DAC), and a detailed Data Protection Impact Assessment (DPIA) for each study. Although the model does not rely on individual informed consent, it includes robust safeguards such as pseudonymization, restricted access, and strict governance. Patients retain the right to exercise their GDPR data rights, and no data is commercialized or shared outside authorized use cases. Future developments may explore structured mechanisms for patient or public involvement in the governance of data access and research prioritization.

While the regional (Joint Resolution 1/2021 allows for the export of health data for EU-funded projects, subject to ethics and DAC approval, PAGEM itself is not designed to facilitate data transfers. Instead, it is optimized for secure *in situ* analysis and federated research. As such, PAGEM is suitable for participating in initiatives like the European Medicines Agency's DARWIN EU initiative (36) and the European Health Data and Evidence Network (EHDEN) (37), where standardized analytical pipelines are deployed locally, and only aggregate, non-sensitive outputs are shared, fully preserving data sovereignty.

In essence, the data access regulation described is completely compliant with the EHDS regulations and would fit perfectly in the proposed future federated structure of the EHDS.

### 3.3 Sample size in region-wide studies

In conventional clinical studies, sample size estimation is critical to ensure statistical power and compliance with data minimization principles. In region-wide retrospective studies using RWD, such as those conducted within PAGEM, the analytical cohort can be defined by all individuals in the target population who meet the inclusion criteria. This exhaustive approach minimizes sampling bias and enhances representativeness. Importantly, data minimization remains a core requirement: the necessity and proportionality of the data collected are evaluated during the study design phase and documented in the DPIA, in accordance with Article 5(1)(c) of the GDPR. Thus, although traditional statistical power calculations may not be required, each study justifies the scope and granularity of the data used as adequate and necessary for the research objectives.

## 4 Results

### 4.1 Evidence generation possibilities

**Figure 2:**
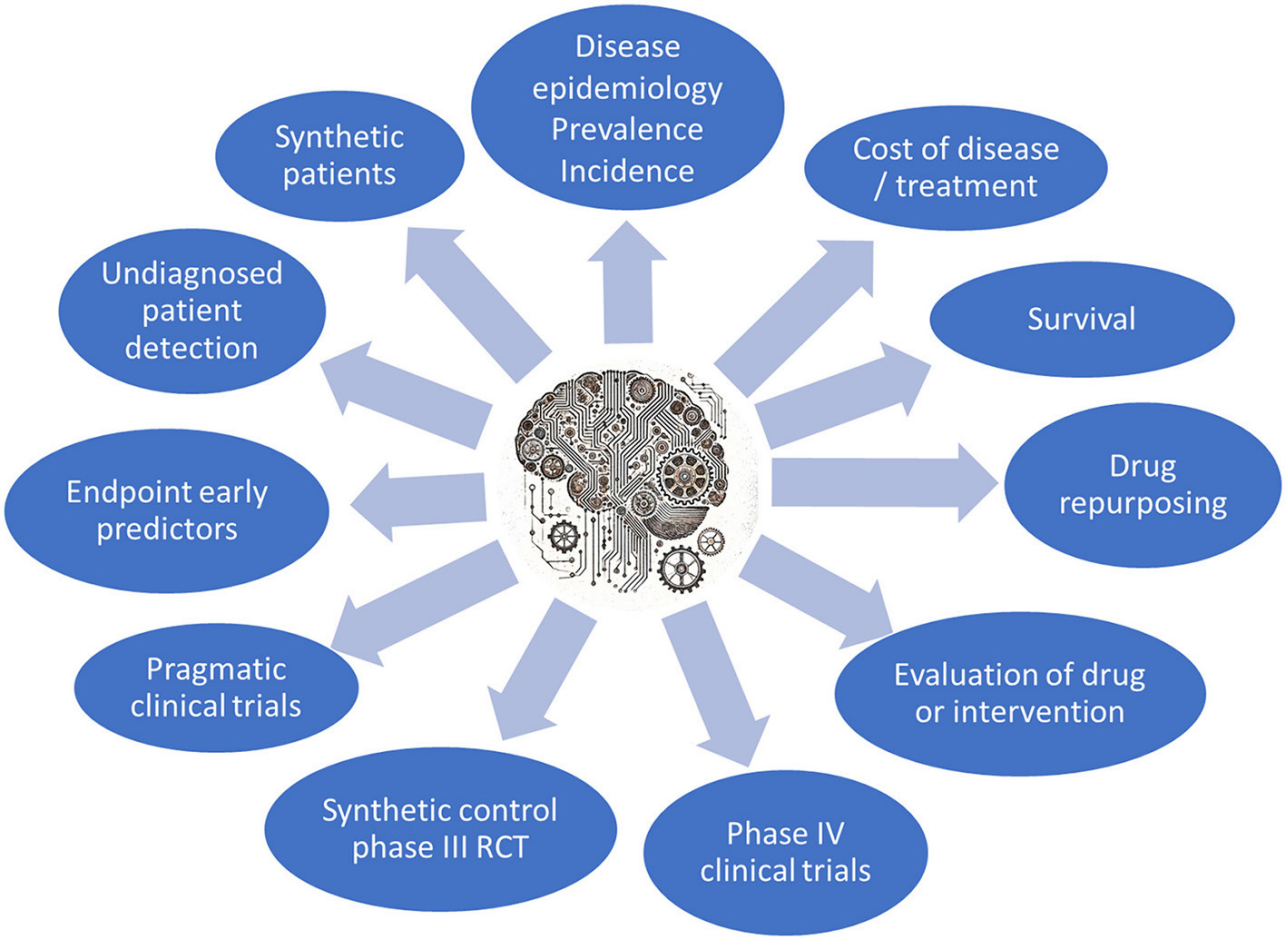
The main types of studies that can be performed in the PAGEM.

Multiple type of studies can be carried out in large RWD repositories. Figure 2 summarizes the most common studies already performed, ongoing or under consideration in the PAGEM infrastructure. Clockwise from the top, the most common studies currently requested by pharma companies are epidemiological studies of prevalence or incidence of diseases (a total of 60%), followed by more detailed studies of cost of disease or interventions (~20%).

Survival studies (38) are fundamental in medical research as they provide crucial insights into patient prognosis, the efficacy of treatments, and the natural course of diseases. These studies allow for the estimation of survival probabilities over time, identifying factors that may influence patient outcomes, such as comorbidities, demographics, and treatment modalities. By analyzing survival data, researchers can also identify potential risk factors and guide clinical decision-making.

Since many patients will present concomitant drug treatments, it is relatively easy to extend the concept of survival studies to repurposing studies by modeling the effect of the concomitant drugs in the outcome of the patient. Similarly, specific treatments or interventions can be assessed, taking into consideration all the possible confusion variables, providing valuable information on the efficacy of these. Actually, in the case of post-marketing surveillance of drugs, they are Phase IV studies (8) of clinical trials.

As previously mentioned, the secure data management environment used here involves the utilization of pseudonymized data, which makes possible patient re-identification, if required by the study and authorized by the ethics committee. Studies aiming to detect undiagnosed patients of rare diseases affected by the well-known diagnostic odyssey (39) or to discover undiagnosed patients of an infectious condition, like Hepatitis C, HIV, etc., for eradication programs (40), or other similar ones, are ultimately oriented to the identification of individuals, which is possible with pseudonymized data but not with anonymized data.

Probably one of the most interesting studies for RWE generation using retrospective cohorts are early predictors of diagnosis, treatment outcomes or other endpoints (9, 10).

Apart from the obvious use of retrospective data in Phase IV studies mentioned above, other innovative applications are also possible in clinical trials. Recruitment and retention of control intervention arm patients, generally consisting of placebo, poses ethical and logistic challenges, especially in oncology (41). Thus, a variety of synthetic control statistical methods can be used to evaluate the comparative effectiveness of an intervention using external control data, defined as cohorts of patients from external sources (42).

Another innovative application of Deep Learning (DL) methods to large datasets is the generation of synthetic data. They can be extremely useful if they meet two conditions: (1) high fidelity, meaning the generated data maintain utility for the intended task, such as yielding comparable performance when training a diagnostic model; and (2) compliance with privacy standards, ensuring that no real patient identities are disclosed in the synthetic dataset (43). Generative adversarial networks (GANs) have been remarkable success, giving rise to diverse generative models for EHR synthesis, oriented to various clinical purposes (44–46). The importance of having access to original RWD to generate high fidelity simulated data is clearly demonstrated by the fact that AI models tend to collapse when trained on recursively generated data (47).

It is worth noting that, unlike in static databases, BPS is updated on a monthly basis, opening thus the possibility of not only retrospective studies, but also prospective or ambispective proposals.

### 4.2 Use cases

PAGEM has successfully been used for carrying out numerous RWE generation studies. Many retrospective studies using large databases of RWD focus on the efficacy of treatments or drugs. In a recent study performed in PAGEM, the evidence of the association between increased use of direct oral anticoagulants and a reduction in the rate of atrial fibrillation-related stroke and major bleeding at the population level was demonstrated using a population of 95,085 patients (48). Actually, the same methodology can be used for different types of interventions, and some original retrospective studies can be carried out. As an example, the Andalusian Genomic Surveillance System (49), specifically the COVID-19 circuit (50), made available a large number of SARS-CoV-2 viral genomes which, in combination with the clinical data of the patients, has been used to carry out an interesting study of the effect of the viral lineage and specific viral mutations on patient survival (51). Another different application of the methodology is the study of the effect of other concomitant pharmacologic treatments in the outcome of the disease. Finding unexpectedly good prognostics associated with other drugs provided by other reasons to the patients can lead to drug repurposing proposals. Some examples of drug repurposing have been made in PAGEM during the recent pandemics. Thus, it has been demonstrated that several drugs, like vitamin D (52) or antipsychotic drugs like aripiprazole have a significant protective effect on COVID-19 patient survival (53). This study was further generalized to discover a significant protective effect in a total of 21 drugs of common use in patients (7).

Another interesting aspect is the identification of the population at risk of different diseases. In the recent COVID-19 pandemics, the identification of individuals at risk of severe infection was a priority for clinicians and health systems, and was successfully addressed using RWD from BPS (54).

Early endpoint predictors are also of paramount interest for any health system. Recently, a model was developed to identify individuals at high risk of ovarian cancer without the need of using specific tumor markers or prior stratification into risk groups using only clinical variables from BPS like demographics, comorbidities, symptoms, blood test results, and healthcare utilization patterns (55).

Recently, as an example of innovative clinical data utilization, ~1 million real electronic health records (EHRs) from diabetic patients were employed to train a GAN, the medGAN (44). The goal was to generate synthetic EHRs that closely mimic the characteristics of diabetic patients while ensuring that the data do not correspond to any actual individuals, thus preserving patient privacy. These data are available within the “Synthetic Clinical Health Records” challenge of the Critical Assessment on Massive Data Analysis conference (56).

### 4.3 Governance and opportunities for collaboration

The PAGEM governance structure ensures that research collaborations align with the strategic priorities of the Andalusian Health System. An internal committee evaluates proposals based on their relevance, resource availability, and financial feasibility. This structured approach maximizes the platform's impact on medical research and innovation.

Currently, PAGEM has established agreements with over 10 major pharmaceutical companies and three Contract Research Organizations (CROs). These partnerships generate revenue that sustains its infrastructure, enabling access for public health initiatives and independent research. This collaborative model fosters medical knowledge generation while maintaining financial sustainability. It is important to note that these collaborations do not involve the sale or transfer of any individual-level data. All data remain securely within the SPE, and analyses are conducted exclusively by authorized personnel from the public health system. External collaborators receive only aggregate results, and all projects, public or private, must be approved by an accredited ethics committee (CCEIBA) and the Data Access Committee. These partnerships are framed as research collaborations aimed at generating public value. In addition to financial sustainability, they contribute to drug safety surveillance, comparative effectiveness research, and development of predictive models, ultimately benefiting the public health system and patient care.

At the international level, PAGEM utilize federated analytical models based on the OMOP common data standard (57), ensuring interoperability and large-scale Real-World Evidence (RWE) generation in federated research networks, like the European Medicines Agency's DARWIN EU initiative (36) and the European Health Data & Evidence Network (EHDEN) (37).

Compared to the EHDS framework, the NHS model in the UK includes centrally managed databases with opt-out provisions for patients (58), while the U.S. Health Insurance Portability and Accountability Act (HIPAA) framework (59) focuses more heavily on institutional data controllers and breach notification mechanisms. EHDS emphasizes federated data access with public oversight, which PAGEM mirrors by embedding analysis within a secure, non-exportable infrastructure.

## 5 Discussion

### 5.1 The future of precision preventive medicine

A key aspect of precision preventive medicine is the development of early endpoint predictors, which anticipate adverse health outcomes before symptoms appear (60). By leveraging machine learning algorithms, healthcare providers can implement targeted interventions, improving patient outcomes and reducing healthcare costs (61). For example, predictive models have been successfully applied to cardiovascular disease (61) and cancer prevention (9, 62).

Most existing prediction models are applied to pre-stratified risk populations, meaning individuals already under clinical monitoring. However, ideal predictors should be applicable to the general population, using routinely collected healthcare data. A notable example is the early predictor for ovarian cancer, developed using RWD from BPS, which identifies high-risk individuals based on clinical variables rather than traditional tumor markers (55). This approach is particularly relevant for high-mortality diseases, where early detection significantly improves survival rates (63).

**Figure 3:**
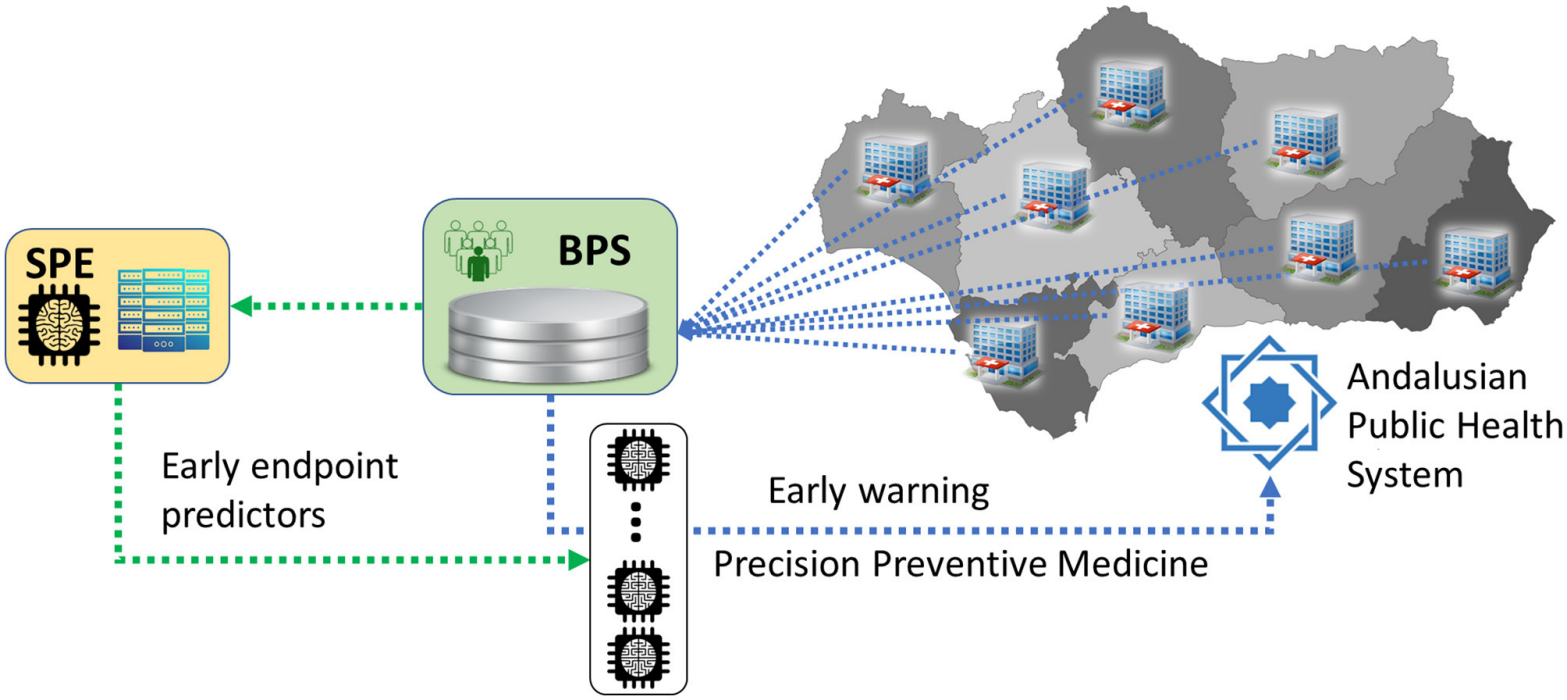
Summarized architecture of the Andalusian Health System with a double layer of digitalization: the first layer, Diraya, for primary use of clinical data for the management of the patient, and a second layer, BPS, for permanent data storage for administrative and research purposes. The Coordinating Ethics Committee (CCEIBA) assesses ethical compliance of each project, while the Data Access Committee (DAC) evaluates the scientific, legal, and proportionality aspects before granting access. The technical architecture includes ingestion from Diraya, structured storage in the BPS, and a secure analytics layer within PAGEM that restricts data access to authorized personnel. The SPE is connected to the BPS in order to promote secondary use of clinical data for research purposes. In particular, predictors developed using BPS data can be run directly over BPS. By doing this, the data repository at the end of the data production chain of the health system becomes the first line in the application of preventive medicine.

Developing robust predictors requires large datasets, computational power, and expertise, but once established, they can be efficiently applied at scale. As illustrated in Figure 3, integrating predictive analytics into healthcare systems can transform them from reactive to preventive models, ensuring earlier interventions and more cost-effective patient management.

### 5.2 Prevention and the future sustainability of the health system

The long-term sustainability of healthcare systems may benefit from shifting toward a predictive and preventive model. Integrating cost-effective endpoint predictors into clinical workflows has the potential to improve patient outcomes while optimizing the use of healthcare resources.

Several studies have shown that the use of Real-World Data (RWD) and predictive models can reduce the cost and duration of evidence generation compared to traditional randomized controlled trials, particularly in post-marketing surveillance and comparative effectiveness research (9, 10). Moreover, public-private research collaborations, as managed by PAGEM, generate revenue that can support infrastructure maintenance and open access for public health studies. While further research is needed to quantify these economic effects, such models represent a promising direction for equitable and data-driven innovation.

A core principle of this model is justice, given that patient data belong to them, and the benefits derived from their use must return to patients (64). By leveraging predictive analytics, data-driven research, and ethical governance, healthcare systems can become self-sustaining knowledge hubs, reinforcing both scientific progress and public wellbeing.

The collaborative model presented here represents a transformative step in healthcare, integrating the PAGEM, a SPE, with the BPS, a large clinical data repository, all within a robust legal framework to drive medical research and innovation. By facilitating an ethical and secure use of RWD, the platform fosters and accelerates the generation of medical evidence.

Although PAGEM is currently the only operational SPE within the Andalusian health system, the regulatory framework (Joint Resolution 1/2021) allows for the creation of additional SPEs if needed for domain-specific or geographically distributed projects. PAGEM does not currently allow remote access by external researchers but supports federated analyses using OMOP-CDM and similar standards. It handles a broad range of data types, including structured EHRs, imaging, lab results, and genomics. Future development plans include expanding analytical toolsets and exploring controlled user access pathways, in alignment with evolving EHDS requirements.

The integration of cost-effective endpoint predictors enhances early intervention, patient outcomes, and healthcare efficiency, particularly in aging populations, shifting healthcare from a reactive to a preventive approach. Additionally, public-private partnerships generate revenue, sustaining infrastructure while ensuring that the benefits of patient data return to society.

By combining data security, regulatory compliance, and large-scale analytics, PAGEM establishes a scalable and replicable model for regional and international healthcare systems. This data-driven approach strengthens healthcare's role in knowledge generation, economic sustainability, and public health improvement, positioning Andalusia as a leading example of ethical, secure, and innovative healthcare digitalization.

## Data Availability

The original contributions presented in the study are included in the article/supplementary material, further inquiries can be directed to the corresponding author.
